# BLOOD PRESSURE MONITORING BY CAREGIVERS: EXAMINING THE INTERSECTION OF ALZHEIMER’S DISEASE AND HYPERTENSION

**DOI:** 10.1093/geroni/igae098.2580

**Published:** 2024-12-31

**Authors:** Dalia Regos-Stewart, Noel Barragan, Renee Fraser, Tony Kuo

**Affiliations:** Los Angeles County Department of Public Health, Los Angeles, California, United States; Los Angeles County Department of Public Health, Los Angeles, California, United States; Fraser Communications, Los Angeles, California, United States; Los Angeles County Department of Public Health, Los Angeles, California, United States

## Abstract

Older adults (OA) experience a high prevalence of comorbid chronic conditions, for which they oftentimes rely on the aid of unpaid caregivers. OA with Alzheimer’s disease (Alzheimer’s) are particularly reliant on these caregivers to monitor their health. For example, hypertension can be highly prevalent in this group, predisposing them to a variety of heart-brain complications. Yet, in spite of emerging evidence of home blood pressure monitoring’s (HBPM’s) effectiveness, caregivers do not always perform nor view this duty as essential. To date, little is known about their experiences with monitoring/managing hypertension. To address this gap in practice, an online panel survey of U.S. caregivers was conducted July-August, 2023. Overall, 600 caregivers (18+ years) of adults (45+ years) participated in the survey; 29% (n=175) provided care to a person with Alzheimer’s. A majority reported monitoring chronic conditions, administering medications, managing medical appointments, and measuring BP. Among those who measure BP, 90% reported feeling confident in their ability to do so accurately; 70% measure it daily/few times a week. A higher proportion of caregivers who work with OA with Alzheimer’s (versus without) reported receiving proper training for measuring BP (50% vs. 45%). The former group reported cultural, language, and transportation barriers to receiving BP monitoring information from health professionals, compared to the latter group (28% vs. 20%). Rich insights about caregivers’ experiences with hypertension management were gleaned from this survey. They suggest opportunities where medicine and public health can further assist caregivers, especially caregivers who are helping OA with Alzheimer’s.

